# Central Serous Chorioretinopathy after Solar Eclipse Viewing

**Published:** 2010-07

**Authors:** Allie Lee, Timothy Y Y Lai

**Affiliations:** Department of Ophthalmology and Visual Sciences, The Chinese University of Hong Kong, China

**Keywords:** Central Serous Chorioretinopathy, Solar Retinopathy, Solar Eclipse, Optical Coherence Tomography (OCT)

## Abstract

**Purpose:**

To report a case of central serous chorioretinopathy after solar eclipse viewing.

**Case Report:**

A middle-age man developed a sudden-onset unilateral scotoma after viewing a partial solar eclipse in Hong Kong. Fundus examination, fluorescein angiography, and optical coherence tomography showed features compatible with central serous chorioretinopathy. The patient was managed conservatively and reevaluated periodically. Serial optical coherence tomographic evaluations demonstrated an initial increase in the amount of subretinal fluid which spontaneously resolved 10 weeks after the onset of symptoms.

**Conclusion:**

This case demonstrates the possibility of development of central serous chorioretinopathy following solar eclipse viewing.

## INTRODUCTION

Retinopathy following viewing of a solar eclipse without the use of safe eyewear has been well-documented in the literature. The usual lesion consists of mild foveal changes due to photochemical damage to the retina. Patients usually present with reduced visual acuity and a central scotoma. Classical findings on fundus examination include a yellow-white spot in the fovea surrounded by granular changes in the retinal pigment epithelium (RPE). Fundus fluorescein angiography (FA) may reveal a small central foveal window defect. Optical coherence tomography (OCT) may also demonstrate hypo- or hyperreflective lesions in the outer retina and RPE.^1^–^4^

Central serous chorioretinopathy (CSCR) is an entity characterized by neurosensory retinal detachment with or without RPE detachment. Previous studies have reported the association of CSCR with various risk factors such as psychological stress, type A personality, pregnancy, untreated hypertension, use of corticosteroids, and psychopharmacologic medications.^5^–^7^

Herein, we describe a rare case of CSCR after viewing a solar eclipse. To our knowledge, the association between CSCR and solar eclipse viewing has not been reported in the literature.

## CASE REPORT

**Figure 1:**
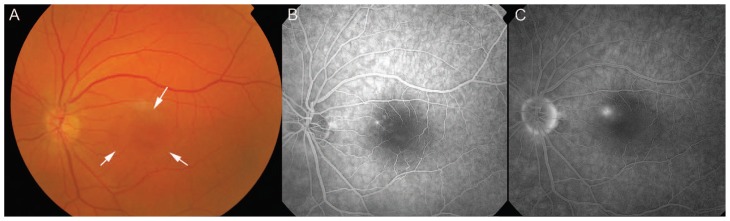
**(A)** Fundus photograph of the left eye showing shallow subretinal fluid accumulation (arrows) involving the fovea with mild pigmentary changes at the level of the retinal pigment epithelium. **(B)** Early phase and **(C)** late phase fluorescein angiography of the same eye showing progressive fluorescein leakage 0.5 disc-diameter in size nasal to the fovea.

**Figure 2:**
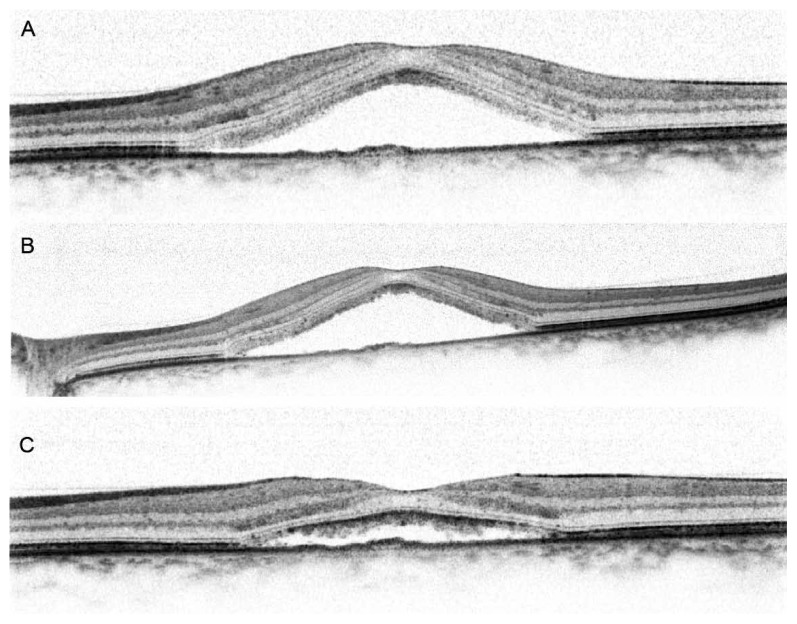
Serial spectral domain optical coherence tomography (OCT) examinations of the left eye. **(A)** On presentation, OCT demonstrated neurosensory retinal detachment with subretinal fluid (central foveal thickness, 552μm). **(B)** Six weeks after the onset of symptoms OCT shows an increase in the amount of subretinal fluid (central foveal thickness, 610μm). **(C)** Ten weeks after the onset of symptoms OCT showing a decrease in subretinal fluid (central foveal thickness, 293μm).

A 44-year-old Caucasian man with good past health and unremarkable ophthalmic history presented with an acute-onset relative scotoma in his left eye after viewing a partial solar eclipse in Hong Kong on July 22, 2009.^8^ The duration of viewing had been brief and less than two seconds. He had not used any protective eyewear while sun gazing and denied the use of corticosteroid medications or any recent stressful event. He presented to the eye clinic two days later with best-corrected visual acuity of 20/20 and 20/18 in his right and left eyes, respectively. Anterior segment examination was unremarkable. Fundus examination revealed shallow subretinal fluid accumulation and RPE changes involving the fovea in the left eye (Fig. 1A). FA showed progressive fluorescein leakage nasal to the fovea, without evidence of choroidal neovascularization (Figures 1B, 1C). Spectral domain OCT scan of the left eye demonstrated serous detachment of the neurosensory retina involving the fovea with central foveal thickness of 552μm (Fig. 2A). No abnormality was detected on FA and OCT in the unaffected fellow eye. A diagnosis of CSCR of the left eye was made and conservative management was adopted.

The patient was reassessed six weeks later and he still complained of a persistent central scotoma. Nonetheless, visual acuity remained stable. Fundus examination of the left eye showed an increase in subretinal fluid. OCT demonstrated an increase in the serous neurosensory retinal detachment (Fig. 2B). Ten weeks after the onset of symptoms, the patient no longer complained of any visual disturbances and best-corrected visual acuity was 20/20 in both eyes. OCT of the left eye showed reduction in the amount of subretinal fluid (Fig. 2C). Considering the spontaneous improvement, conservative management was continued.

## DISCUSSION

To the best of our knowledge, this is the first report describing acute CSCR after direct solar eclipse gazing. Unprotected sun-gazing, particularly during an episode of solar eclipse, is a well-known cause of solar retinopathy.^1^–^4^ Classically, patients present with decreased visual acuity and a central scotoma, together with fundoscopic findings consisting of a yellow-white spot in the fovea surrounded by granular RPE changes.^2^ Typical OCT findings in solar retinopathy include hypo- or hyperreflective lesions in the outer retina and RPE.^3^ None of the reports on solar retinopathy have noted the presence of subretinal fluid as in our case.

Clinical features of CSCR include accumulation of subretinal fluid, neurosensory retinal and RPE detachment, and leakage with angiographic evidence of RPE hyperpermeability.^5^ Retrospective case series and case-control studies have identified various systemic risk factors associated with CSCR including psychological stress, type A personality, pregnancy, untreated hypertension, corticosteroids, and psychopharmacologic medications.^6^,^7^ However, our patient reported none of these risk factors. In addition, the clear temporal relationship between exposure and onset of symptoms demonstrated that CSCR was most likely a direct consequence of solar damage.

The exact pathogenetic mechanism of CSCR in our case remains unclear. We hypothesize that intense sunlight was preferentially absorbed by melanosomes in the RPE, causing localized RPE damage and thus resulting in CSCR.^9^ This may be similar to experimental serous retinal detachment caused by intense light over damaged RPE.^10^ In our patient, the photoreceptors were relatively intact due to brief sunlight exposure and visual acuity was largely preserved. Since no previous case has been reported in the literature, the patient described herein simply demonstrates the possibility of CSCR after viewing a solar eclipse; further cases are needed to draw a definitive conclusion on the causal relationship between the two.
